# Extracellular Vesicles and the Gartner Hype Cycle

**DOI:** 10.1002/jex2.70104

**Published:** 2025-12-19

**Authors:** Mahsa Salehi, Shukoofeh Torabi, Homeyra Seydi, Faezeh Shekari, Massoud Vosough

**Affiliations:** ^1^ Biological Products and Blood Safety Research Center High Institute for Research and Education in Transfusion Medicine Tehran Iran; ^2^ Department of Regenerative Medicine, Cell Science Research Centre Royan Institute for Stem Cell Biology and Technology ACECR Tehran Iran; ^3^ Anatomical Sciences Research Centre, Institute for Basic Sciences Kashan University of Medical Sciences Kashan Iran; ^4^ Advanced Therapy Medicinal Product Technology Development Centre (ATMP‐TDC), Cell Science Research Centre, Royan Institute for Stem Cell Biology and Technology ACECR Tehran Iran; ^5^ Department of Stem Cells and Developmental Biology, Cell Science Research Centre, Royan Institute for Stem Cell Biology and Technology ACECR Tehran Iran; ^6^ Experimental Cancer Medicine, Institution for Laboratory Medicine Karolinska Institute Stockholm Sweden

## Abstract

Extracellular vesicles (EVs) have gained significant attention as emerging tools in diagnostics and therapeutics. Using the Gartner Hype Cycle framework, this commentary examines the current trajectory of EV research, from initial enthusiasm to growing concerns about reproducibility, standardization and clinical translation. We highlight key challenges, including EV heterogeneity, methodological inconsistencies and publication bias, which risk stalling progress. Ongoing efforts by the International Society for Extracellular Vesicles (ISEV), including Minimal information for studies of extracellular vesicles (MISEV) guidelines and the extracellular vesicle‐transparent reporting and centralizing knowledge (EV‐TRACK) database, have been crucial for advancing the field. We tackle actionable priorities to support rigorous, transparent and clinically meaningful EV research that would prompt the actual translation.

## 1 Extracellular Vesicles and the Gartner Hype Cycle Framework

**FIGURE 1:**
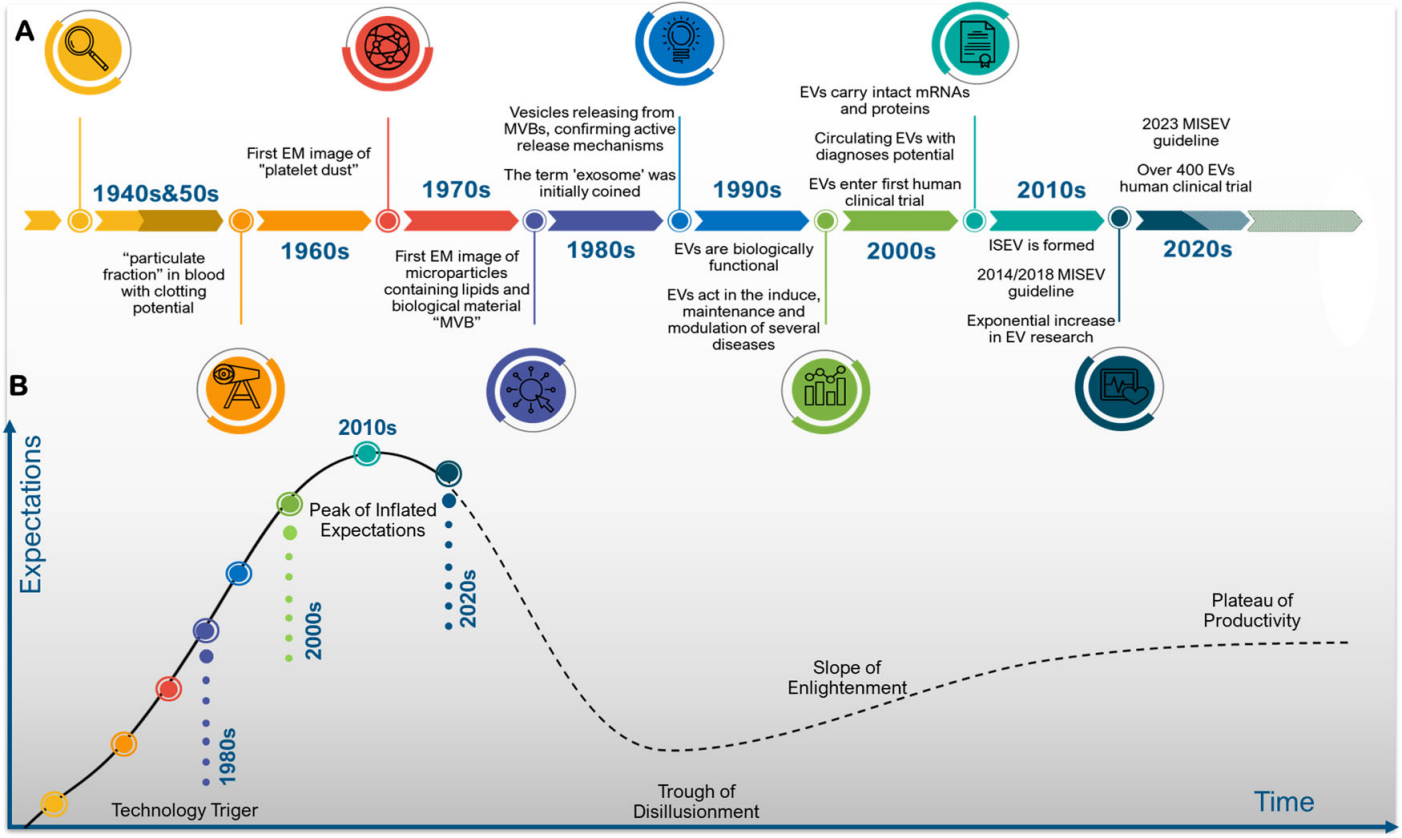
The timeline of EV discoveries and research. (A) The first studies on EVs as biological particles launched in the early 1940s. They were initially considered cellular waste. However, in the early 1980s, investigations provided deeper insights into their structure, composition and biomedical functions. In the 2010s, the establishment of the ISEV marked a turning point in the global perspective of EV research, encouraging greater methodological alignment. After launching MISEV 2018 and MISEV 2023, we can see more compliance with the mentioned guidelines in publications and study designs. (B) Gartner Hype Cycle schematically depicts the maturity of EV‐based innovations in a timeline. The peak of inflated expectations was in the 2010s. Although current studies highlight new trends and emerging EV technologies, ongoing challenges in EVs isolation, validation practices and the approval process may drive the field toward the ‘Trough of Disillusionment’. EM, electron microscopy; EVs, extracellular vesicles; ISEV, international society for extracellular vesicles; MISEV, minimal information for studies of extracellular vesicles; MVBs, multivesicular bodies.

The study of extracellular vesicles (EVs), membrane‐bound particles released by various cells that carry proteins, lipids and nucleic acids, represents a rapidly evolving field in biomedical research. As shown in Figure 1, their historical timeline dates back to the early 1940s, when Chargaff and West described a ‘particulate fraction’ in blood with high clotting potential (Pan and Johnstone 1983). Subsequent extensive research has revealed that, despite initial perceptions of EVs as a waste disposal system, they function as a sophisticated cell‐to‐cell communication tool, playing crucial roles in various pathophysiological conditions (Pan and Johnstone 1983; Rai et al. 2024).

To better understand the trajectory of EV research and its translation into practice, the Gartner Hype Cycle provides a useful lens for examining the evolution of enthusiasm, scepticism and eventual maturation in the field. The Gartner Hype Cycle is a conceptual framework that describes the maturation and adoption of emerging technologies through five distinct phases: (1) Technology Trigger, early proof‐of‐concept sparks interest and initial development; (2) Peak of Inflated Expectations, enthusiasm generates unfounded excitement and unrealistic expectations; (3) Trough of Disillusionment, interest declines as early implementations fail to meet expectations; (4) Slope of Enlightenment, the technology's benefits become more widely understood and viable applications begin to emerge; and (5) Plateau of Productivity; the technology achieves mainstream adoption and delivers stable, proven benefits. This model is useful for understanding how scientific innovations gain attention, face scepticism and eventually find stable, meaningful applications after being tested for reproducibility and practical applications.

If we imagine the EV positioning within the Gartner Hype Cycle, growing interest in their roles as biomarkers and as novel therapeutic tools across last decades would be in line with the early to mid‐stages of the technology (Couch et al. 2021). EVs have attracted substantial scientific and commercial interest, leading to a surge in clinical trials and discussions around their potential as next‐generation diagnostics and therapeutics. Today, more than 400 EV‐based clinical trials are underway, positioning EVs on the cusp of becoming a next‐generation, theranostic platform with the potential to revolutionize modern medicine (Mizenko et al. 2024), fuelling widespread enthusiasm, characteristic of the Peak of Inflated Expectations. However, as seen in other transformative technologies such as CRISPR‐Cas9 gene editing, Initial excitement often precedes the development of robust validation procedures and universally accepted protocols (Intemann 2022; Nissen et al. 2016; Flier 2022). Unfortunately, irreproducible data whether due to technical limitations or errors, lack of rigor, unappropriated study design or publication bias, can accelerate the descent into the ‘Trough of Disillusionment’. Beyond academic enthusiasm, the commercialization of EV‐based products is also beginning to take shape, reflecting both the promise and the challenges of translating EV technologies into clinical practice.

## 2 EVs Commercialization, Hype or Hope

As a paradigm of cellular communication, EVs have opened a new window for theranostics (Greening et al. 2025). In this regard, they have garnered significant attention as powerful diagnostic tools with high accuracy and sensitivity, as they reflect the status of their parent cells and can be isolated from other components (Cheng and Kalluri 2023). The ExoDx Prostate IntelliScore (EPI) test was the first EV‐based diagnostic assay to receive FDA breakthrough device recognition, after its clinical performance was validated across multiple studies for over 7 years (Tutrone et al. 2023; Margolis et al. 2022; Kretschmer et al. 2022; Tutrone et al. 2020). This designation has since been extended to the Mercy Halo Ovarian Cancer Test (OC Test), developed for screening ovarian cancer in asymptomatic postmenopausal women (Winn‐Deen et al. 2024) and to Mursla Bio's EvoLiver for the early detection of hepatocellular carcinoma (Greening et al. 2025). Regarding therapeutics, EVs also hold immense potential, prompting some biopharmaceutical companies to focus on their development (Cheng and Kalluri 2023). However, several challenges, including EVs heterogeneity, limited pharmacokinetic understanding, scalability issues in manufacturing and a lack of robust clinical data, hinder their regulatory approval (Greening et al. 2025). Furthermore, the successful clinical translation of EV‐based therapeutics requires sustained funding, particularly to support the generation of long‐term safety data. However, limited commercial interest and inadequate financial support have created a Catch‐22 situation: without adequate data, investment remains scarce and without investment, the data needed to validate these therapies cannot be produced. This dynamic both contributed to and has been further supported by the non‐sustainability of pioneering companies such as Codiak Biosciences (Lee 2024). The absence of a dedicated regulatory framework for EV‐based therapeutics further compounds these issues. In response, the ISEV has recently established a ‘Regulatory Affairs Task Force’, which, alongside other task forces and cross‐society working groups, aims to support translational efforts by identifying applicable regulatory guidance and facilitating its implementation in EV‐based clinical research (https://www.isev.org/taskforces). It seems that continued financial investment, rigorous scientific validation and close collaboration among researchers, clinicians, industry leaders and regulatory authorities are key driving forces in accelerating the successful translation of EV‐based therapeutics moving from promise to practice (Cheng and Kalluri 2023; Wang et al. 2024).

## 3 Emerging Challenges: Reproducibility, Heterogeneity and Publication Bias

Despite expanding interest, various challenges continue to undermine the field's credibility and practical progress. One of the primary obstacles is EV heterogeneity, which complicates their isolation, characterization and functional assessment (Welsh et al. 2024). Variability in EV size, density and molecular cargo affects reproducibility, making it difficult to compare results across different studies and laboratories (Welsh et al. 2024; Carney et al. 2025). Pre‐analytical factors such as sample collection, processing and storage conditions also influence EV quantity, quality and functional properties (Welsh et al. 2024). Moreover, EVs isolated from the same source using different isolation strategies exhibit variations in content and function, further compounding heterogeneity‐related challenges (Welsh et al. 2024; Abyadeh et al. 2024; Kashani et al. 2023; Eslami et al. 2023). Additional purification steps to eliminate protein contaminants can also affect experimental outcomes and lead to data misinterpretation.

Additionally, methodological variability can lead to false‐positive findings. For example, identifying EV‐associated biomarkers that fail to reproduce across cohort studies or experimental setups may hinder clinical translation, leading to misdiagnosis, inadequate patient stratification, or ineffective therapy protocols. Moreover, because EVs are investigated across a wide range of diseases with varying prevalence, the risk of false positives becomes particularly pronounced in low‐prevalence conditions, even when test sensitivity and specificity remain constant. Altogether, these complexities have led to reduced reproducibility and inconsistent results across studies.

Studies on EVs and related technologies reveal a typical pattern of emerging biomedical innovations that begins with initial excitement and hype, followed by growing doubts. However, the field appears to be transitioning from the ‘Peak of Inflated Expectations’ to the ‘Trough of Disillusionment’ (Figure 1). Key challenges, such as the establishment of standard isolation methods, ensuring methodological coherence, and proposing validation protocols, are essential steps in the advancement of this phase. These challenges should not be considered as potential obstacles in the field but rather as integral components of progress that necessitate refining current methodologies, validating the reproducibility of procedures, and emphasizing clinical applications. To gain a more realistic understanding of EV potential, recent efforts have focused on improving characterization techniques, developing good manufacturing practice (GMP) compatible processes and fostering international collaboration on the globalization of standard protocols and methodologies.

## 4 Standardization and Rigor: Moving Toward the Slope of Enlightenment

As the largest community dedicated to EVs, the International Society for Extracellular Vesicles (ISEV) was established in 2012 (Royo et al. 2020; Raposo and Stahl 2023) to systematically guide diverse research findings and incorporate expert insights in the field, with nearly 2000 members (www.isev.org). Members of this society developed a guideline in 2014, which was subsequently updated in 2018 and 2023 (Welsh et al. 2024; Lotvall et al. 2014; Théry et al. 2018). The primary objective of these guidelines is to establish consensus‐based principles for the nomenclature of vesicles, address challenges related to their isolation, characterization and storage, and recommend appropriate tools and methods based on available resources. Ultimately, these efforts aim to enable researchers to conduct credible studies with reproducible results in the EV field (Welsh et al. 2024; Théry et al. 2018, Lötvall et al. 2014). Furthermore, to promote reproducibility and a culture of transparent and careful reporting in EV studies, researchers register their detailed EV methodology experiments in EV‐transparent reporting and centralizing knowledge (EV‐TRACK), an open‐access database endorsed by ISEV (https://www.isev.org/taskforces; www.evtrack.org). Adherence to these guidelines has been reported to positively impact the EV community and significantly strengthen methodological quality (Poupardin et al. 2021).

Beyond standardization and guidance, ISEV functions as a vital hub for collaboration and knowledge sharing. The society hosts annual congresses, conferences, workshops and webinars that bring together researchers from various disciplines, promoting interdisciplinary problem‐solving (www.isev.org). Various assemblies of eager researchers in the field of EVs, such as the Student Network on EVs (SNEV), massive open online courses (MOOCs), EV clubs and the ISEV annual meeting, further foster community engagement. ISEV‐related publications, including statements and position papers, offer clarity and consensus on best practices, enabling laboratories worldwide to generate reliable and comparable data (Royo et al. 2020). Therefore, ISEV's efforts focus on promoting more rigorous and precise EV research.

Simultaneously, as validation and safety studies advance, attention is increasingly turning toward clinical translation. With ongoing improvements in regulatory frameworks and quality standards, EV technologies, like other advanced disciplines in science, are poised to reach the ‘Plateau of Productivity’.

For the EV translational ecosystem, including researchers, industry counterparts and policy makers, to advance toward the slope of enlightenment and ultimately reach the plateau of productivity, several priorities are proposed to be addressed (Table 1):

- Rigorous experimental design, including appropriate negative and positive controls.
- Transparent data reporting and sharing of raw datasets for independent verification.
- Adherence to approved international guidelines, such as those proposed by the ISEV, including Minimal information for studies of extracellular vesicles (MISEV) 2018 and MISEV2023.
- Taking advantage of multidisciplinary approaches, combining biophysics, omics technologies and bioinformatics to unravel the complexity of EV biology.

**TABLE 1:** Current challenges and possible solutions in EV research.

Category	Challenges	Impact of related challenges	Recommended solutions
Intrinsic EV heterogeneity	Individual cells release multiple EV subtypes with varying size, density and cargo composition	Inconsistent functional outcomes; conflicting findings; reduced reproducibility across studies	Detailed reporting of EV isolation and characterization parameters; avoid overinterpretation and unsupported EV nomenclature
EV isolation methods	Lack of standardized protocols across different isolation techniques	Variability in purity and yield; contamination risks; inconsistent downstream results	Adoption of transparent reporting per MISEV guidelines and EV‐TRACK recommendations
EV characterization	Presence of co‐isolated components; absence of a universal quantification method; lack of subtype‐specific markers and validated commercial antibodies	Poor reproducibility and comparability between studies	Comprehensive characterization following MISEV guidelines
Data interpretation	Use of varied, non‐standard EV terminology; overreliance on preliminary or correlative data	Misleading or conflicting interpretations; irreproducibility across studies	Adoption of MISEV‐recommended nomenclature; emphasize blinded validation and independent replication
Publishing pressures	Bias toward positive or novel findings	Skewed scientific literature; difficulty identifying robust or reproducible findings	Promote transparency; encourage publication of negative or null results

**Abbreviations**: EV, extracellular vesicle; EV‐TRACK, extracellular vesicle‐transparent reporting and centralizing knowledge; MISEV, minimal information for studies of extracellular vesicles.

The current challenges posed by unreliable data necessitate global efforts to ensure reproducibility, transparency and rigorous validation. By aligning EV research with these principles, the field can reach its full potential in advancing personalized medicine, improving diagnostics and unlocking new therapeutic strategies.

## Author Contributions

**Mahsa Salehi**: writing – review and editing, writing – original draft, investigation. **Shukoofeh Torabi**: investigation, writing – review and editing. **Homeyra Seydi**: investigation, writing – review and editing. **Faezeh Shekari**: conceptualization, supervision, writing – review and editing. **Massoud Vosough**: conceptualization, supervision, writing – review and editing.

## Funding

This work received no specific grant from any funding agency.

## Conflicts of Interest

The authors declare no conflicts of interest.

## Data Availability

Data sharing is not applicable to this article as no datasets were generated or analysed during the current study.
